# Hyponatremia normalization as an independent prognostic factor in patients with advanced non-small cell lung cancer treated with first-line therapy

**DOI:** 10.18632/oncotarget.13372

**Published:** 2016-11-15

**Authors:** Rossana Berardi, Matteo Santoni, Thomas Newsom-Davis, Miriam Caramanti, Silvia Rinaldi, Michela Tiberi, Francesca Morgese, Mariangela Torniai, Mirco Pistelli, Azzurra Onofri, Marc Bower, Stefano Cascinu

**Affiliations:** ^1^ Clinica di Oncologia Medica, Università Politecnica delle Marche, Azienda Ospedaliero-Universitaria Ospedali Riuniti Umberto I–GM Lancisi–G Salesi, Ancona, Italy; ^2^ Chelsea & Westminster Hospital, London, United Kingdom; ^3^ Oncologia Medica, Università degli studi di Modena e Reggio Emilia, Modena, Italy

**Keywords:** hyponatremia, lung cancer, platinum-base chemotherapy, prognosis, targeted therapy

## Abstract

The aim of the study was to assess, for the first time, the prognostic role of hyponatremia and sodium normalization in patients receiving first-line chemo- or targeted therapy for advanced non-small cell lung cancer.

Four hundred thirty-three patients with advanced non small cell lung cancer were treated with first line chemo- or targeted therapy between 2006 and 2015 at our institutions. Patients were stratified in two groups, with or without hyponatremia (group A and B, respectively). Progression free survival (PFS) and overall survival (OS) were estimated using Kaplan-Meier method. A *Cox regression model was carried out* for *univariate* and *multivariate analyses*.

Sixty-nine patients (16%) presented with hyponatremia at the start of first-line therapy. The median OS was 8.78 months in Group A and 15.5 months in Group B (*p* < 0.001), while the median PFS was 4.1 months and 6.3 months respectively (*p* = 0.24). In Group A, median OS was significantly higher in patients who normalized their sodium levels (11.6 vs. 4.7 months, *p* = 0.0435). Similarly, the median PFS was significantly higher in patients who normalized their sodium levels (6.7 vs. 3.3 months, *p* = 0.011). At multivariate analysis, sodium normalization was an independent prognostic factor for both OS and PFS.

Sodium normalization during first-line therapy is an independent prognostic factor for OS and PFS in patients with advanced lung cancer treated with first-line therapies. Frequent clinical monitoring and prompt treatment of hyponatremia should be emphasized to optimize the outcome of these patients.

## INTRODUCTION

Hyponatremia is a common electrolyte disorder in cancer patients, particularly in those who are hospitalized [1]. Although patients with hyponatremia are often asymptomatic, when symptoms do occur they are mainly neurological and include headaches, lethargy, poor concentration, confusion, vomiting, hallucinations and even coma [2]. Mild chronic hyponatremia can lead to marked gait instability, falls, fractures and a higher incidence and duration of hospitalization [3].

The incidence and prevalence of hyponatremia vary depending on the tumor type, clinical setting, and serum sodium cut-off level [4]. The frequency of hyponatremia was estimated at up to 40% in hospitalized patients and 15% in patients with small cell lung cancer (SCLC) [5, 6]. The syndrome of inappropriate anti-diuretic hormone secretion (SIADH) is the main cause of hyponatremia in malignancy [7], with other causes including heart failure, nephritic syndrome, extracellular volume depletion, chemotherapy [5] and target therapies [8]. The onset of hyponatremia has been associated with worst prognosis in several cancers including SCLC, mesothelioma, renal cell carcinoma, gastrointestinal cancer and lymphoma [9, 10, 11, 12, 13, 14, 15, 16]. Furthermore hyponatremia represents a prognostic factor in terminal cancer patients [17], although a prompt correction of serum sodium level is associated to a longer survival and an improvement of clinical condition [18]. Non-small-cell lung cancer (NSCLC) is a poor-prognosis malignancy, which is the leading cause of cancer related death. Often asymptomatic in the early stages, more than half of patients have metastatic disease at time of first diagnosis [19]. The incidence of hyponatremia in NSCLC varies from 1% to 50% [20]. Early recognition and a prompt treatment of this electrolytic imbalance could prevent clinical complications and improve survival [21].

In this study, we aimed to investigate for the first time the prognostic significance of hyponatremia normalization in patients with advanced NSCLC treated with first line therapy.

## RESULTS

### Patient characteristics

Five hundred and twenty-one patients were treated with first-line therapies at our institutions. Of these, 433 patients (299 males and 134 female) were included in this analysis, whilst 88 were excluded due to lack of complete data.

The median age was 66 years (range 25−86) and the majority were current or former smokers (364 patients, 84%). Histology was adenocarcinoma in 278 patients (64%), squamous carcinoma in 101 patients (23%) and other histology in 54 patients (13%). Tumor stage was III in 112 (26%) patients and IV in 321 patients (74%). Most patients (405, 94%) received first-line chemotherapy whilst 28 (6%) an epidermal growth factor receptor (EGFR) tyrosine kinase inhibitor.

Sixty-nine patients (16%) presented with hyponatremia at the start of first-line therapy (Group A), and 368 patients (85%) were eunatremic (Group B). There were no significant differences in terms of clinicopathological characteristics between the groups (Table 1). Among those in group A, 9 patients (13%) had grade ≥ 2 hyponatremia. Forty-one patients (59%) normalized their serum sodium levels during treatment. Thirteen (18%) patients received saline solution with hyponatremia resolution in 8 (12%) patients, 9 (13%) patients underwent fluid restriction with serum sodium normalization in 4 (6%) patients. Three (4%) patients were suffering from SIADH.

**Table 1:** Patients’ characteristics

Patients	Overall 433 (%)	Serum sodium ≥ 136 364 (84)	Serum sodium ≤ 135 69 (16)	*p*
**Gender**				
Male	299 (69)	250 (69)	49 (71)	*0.77*
Female	134 (31)	114 (31)	20 (29)	
**Age, years**	66	66	67	
Range	25−86	25−85	45−86	
**ECOG-PS ≥ 2**	41 (9)	33 (9)	8 (12)	*0.50*
**ECOG-PS < 2**	392 (91)	331 (91)	61 (88)	
**Histology**				
Adenocarcinoma	278 (64)	238 (65)	40 (58)	*0.15*
Squamous carcinoma	101 (23)	80 (22)	21 (30)	
Other	54 (13)	46 (13)	8 (12)	
**Tumor Stage**				
Stage III	112 (26)	96 (26)	16 (23)	*0.65*
Stage IV	321 (74)	268 (74)	53 (77)	
**EGFR mutation status**				
Wild-type	388 (90)	324 (89)	64 (93)	*0.10*
Mutated	45 (10)	40 (11)	5 (7)	
**Smoking history**				
Former/current smoker	364 (84)	302 (83)	62 (90)	*0.20*
Never smokers	69 (16)	62 (17)	7 (10)	
**Common sites of metastasis**				
Lung	150 (35)	123 (34)	27 (39)	*0.30*
Bone	117 (27)	102 (28)	15 (22)	
Nervous system	68 (16)	62 (17)	6 (9)	
Liver	54 (13)	47 (13)	7 (10)	
**First-line therapy**				
Platinum-based chemotherapy	348 (80)	289 (79)	59 (86)	*0.18*
Non platinum-based	57 (13)	48 (13)	9 (13)	
EGFR- tyrosine kinase inhibitor	28 (7)	27 (8)	1 (1)	
**Response to first-line therapy**				
Partial response	162 (37))	142 (39)	21 (30)	*0.19*
Stable disease	113 (26)	91 (25)	24 (35)	
Progression disease	158 (37)	135 (37)	24 (35)	

### Overall Survival (OS)

Median OS from first-line therapy was 13.4 months (95% CI 11.4 to 15.9) in the overall population. Two hundred and eighty one patients (64.9%) died during their follow-up.

Median OS was 18.2 months (95% CI 15.1 to 27.2) and 13.0 months (95% CI 10.7 to 15.9) in non-smokers and smokers respectively (*p* = 0.21). Stratified by gender, median OS was 12.7 months (95% CI 10.5 to 16.5) in males and 16.2 months (95% CI 12.2 to 25.7) in females (*p* = 0.031). No significant difference was found between patients aged < 70y *vs*. ≥ 70y (14.4 *vs*. 13.0 months, *p* = 0.22). Patients with worse performance status (PS ≥ 2) had a shorter OS compared to those with < 2 (7.2 *vs*. 14.7 months, *p* = 0.001).

Based on histology, the median OS was 13.0 (95% CI 9.5 to 17.5) in patients with squamous carcinoma, 14.4 (95% CI 11.6 to 20.2) in patients with adenocarcinoma and 12.7 (95% CI 9.6 to 16.8) in patients with other histologies (*p* = 0.371). As for EGFR status, patients with EGFR wild-type tumors showed a worst OS compared to mutated tumors (12.7 *vs*. 20.9 months, *p* = 0.03).

**Figure 1:**
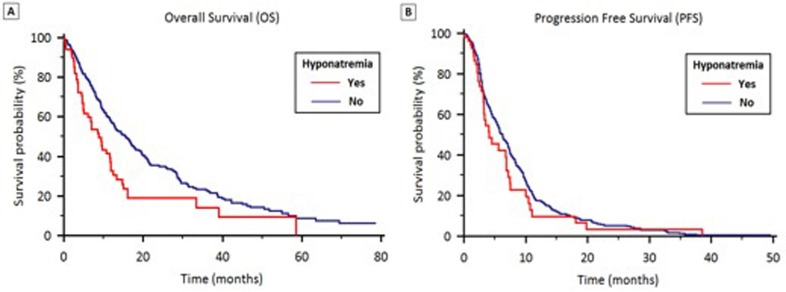
OS (1A) and PFS (1B) stratified by the presence of hyponatremia in patients treated with first-line therapy for locally advanced or metastatic NSCLC

Stratified by hyponatremia, median OS was 8.8 months (95% CI 6.3 to 12.7) and 15.5 months (95% CI 12.4 to 25.1) in groups A and B respectively (*p* < 0.001) (Figure 1A).

**Figure 2:**
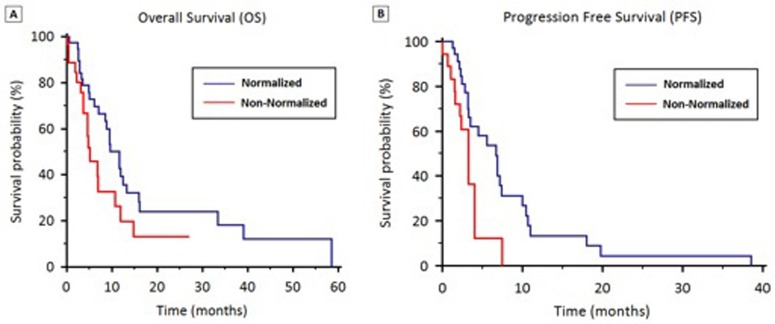
OS (2A) and PFS (2B) stratified by hyponatremia normalization during first-line therapy

For patients with hyponatremia (group A), median OS was significantly higher in patients who normalized their sodium levels (11.6 *vs*. 4.7 months, *p* =0.0435) (Figure 2A).

### Progression-Free Survival (PFS)

In the overall study population, median PFS was 5.9 months (95% CI 3.9 to 7.8). Stratified by gender, median PFS was 5.4 months (95% CI 5.0 to 6.1) in males and 6.9 months (95% CI 5.9 to 8.4) in females (*p* = 0.11).

The median PFS was 6.9 months (95% CI 5.0 to 9.8) and 5.8 months (95% CI 5.3 to 6.5) in non-smokers and smokers (*p* = 0.49). No significant difference in PFS was found according to aged (< 70y *vs*. ≥ 70y, 5.8 *vs*. 6.2 months, *p* = 0.96), performance status (ECOG-PS ≥ 2 *vs*. < 2, 4.0 *vs*. 6.3 months, *p* = 0.14), or histology (squamous carcinoma *vs*. adenocarcinoma *vs*. other histologies: 5.6 *vs*. 6.3 *vs*. 4.2 months, respectively). Patients with EGFR wild-type tumors had a worse PFS compared to those with mutated tumors (5.6 *vs*. 9.4 months, *p* = 0.02).

Stratified according to hyponatremia, median PFS was 4.1 months (95% CI 3.7 to 4.7) in Group A and 6.3 months (95% CI 5.3 to 8.1) in Group B (*p* = 0.24) (Figure 1B). In Group A, median PFS was significantly higher in patients who normalized their sodium levels (6.7 *vs*. 3.3 months, *p* = 0.011) (Figure 2B).

### Univariate and Multivariate analyses in the overall study population

Univariate analysis demonstrated that male gender, PS ≥2, tumor stage IV, non-adenocarcinoma histology, wild-type EGFR status and hyponatremia were significantly associated with worse OS (Table 2A). At multivariate analysis, PS, tumor stage, and hyponatremia were predictors of OS (Table 2A).

**Table 2A:** Univariate and multivariable analysis of predictors of OS in patients treated with first-line therapy for locally advanced or metastatic NSCLC

OVERALL SURVIVAL IN THE OVERALL POPULATION				
	Univariate Cox Regression		Multivariable Cox regression	
	HR (95%CI)	*p-value*	HR (95%CI)	*p-value*
Age (≥ 70y *vs*. < 70y)	1.16 (0.92−1.47)	0.222		
Gender (F *vs*. M)	0.75 (0.58−0.97)	**0.032**	0.77 (0.59-1.00)	0.052
ECOG-PS (≥ 2 *vs*. < 2)	1.88 (1.27−2.78)	**0.002**	1.53 (1.00-2.32)	**0.048**
Smoke status (N *vs*. Y)	0.82 (0. 06−1.12)	0.211		
Tumor Stage (IV *vs*. III)	1.57 (1.20−2.05)	**0.001**	1.70 (1.29-2.24)	**<0.001**
Histology (AC *vs*. non-AC)	0.83 (0.65−1.06)	**0.131**	0.81 (0.63-1.03)	0.090
EGFR Status (MT *vs*. WT)	0.63 (0.42−0.97)	**0.036**	0.74 (0.48-1.14)	0.177
Hyponatremia (Y *vs*. N)	1.71 (1.25−2.34)	**<0.001**	1.59 (1.14-2.21)	**0.006**

Significant values are reported in bold.

AC = Adenocarcinoma; CI = confidence interval; ECOG-PS = Eastern Cooperative Oncology Group Performance Status; EGFR = Epidermal growth factor receptor; F = female; HR = hazard ratio; M = male; MT = mutated status; WT = wild-type status

With respect to PFS, univariate analysis showed that male gender, PS ≥ 2, tumor stage IV and wild-type EGFR status were significantly associated with worse PFS (Table 2B). Multivariate Cox regression analysis revealed that tumor stage IV and wild-type EGFR status were independent prognostic factors for worse PFS (Table 2B).

**Table 2B:** Univariate and multivariable analysis of predictors of PFS in patients treated with first-line therapy for locally advanced or metastatic NSCLC

PROGRESSION-FREE SURVIVAL IN THE OVERALL POPULATION				
	Univariate Cox Regression		Multivariable Cox regression	
	HR (95%CI)	*p-value*	HR (95%CI)	*p-value*
Age (≥ 70y *vs*. < 70y)	0.99 (0.79−1.25)	0.964		
Gender (F *vs*. M)	0.82 (0.64−0.99)	**0.111**	0.83 (0.64−1.08)	0.171
ECOG-PS (≥ 2 *vs*. < 2)	1.39 (0.90−2.14)	**0.139**	1.26 (0.80−1.97)	0.316
Smoke status (N *vs*. Y)	0.90 (0.67−1.21)	0.486		
Tumor Stage (IV *vs*. III)	1.36 (1.05−1.75)	**0.020**	1.41 (1.09−1.83)	**0.010**
Histology (AC *vs*. non-AC)	0.91 (0.72−1.15)	0.433		
EGFR Status (WT *vs*. MT)	0.62 (0.42−0.92)	**0.018**	0.63 (0.42−0.95)	**0.027**
Hyponatremia (Y *vs*. N)	1.23 (0.87−1.73)	0.245		

Significant values are reported in bold.

AC = Adenocarcinoma; CI = confidence interval; ECOG-PS = Eastern Cooperative Oncology Group Performance Status; EGFR = Epidermal growth factor receptor; F = female; HR = hazard ratio; M = male; MT = mutated status; WT = wild-type status

### Univariate and Multivariate analyses in patients with hyponatremia (Group A)

Univariate and multivariate analysis showed that tumor IV disease and failure to normalize sodium neutralization were significantly associated with worse OS (Table 3A).

**Table 3A:** Univariate and multivariable analysis of predictors of OS in the 69 patients with hyponatremia at the start of first-line therapy for locally advanced or metastatic NSCLC

OVERALL SURVIVAL IN HYPONATREMIC PATIENTS				
	Univariate Cox Regression		Multivariable Cox regression	
	HR (95%CI)	*p-value*	HR (95%CI)	*p-value*
Age (≥ 70y *vs*. < 70y)	1.18 (0.64−2.20)	0.598		
Gender (F *vs*. M)	0.99 (0.54−1.84)	0.990		
ECOG-PS (≥ 2 *vs*. < 2)	1.29 (0.62−2.67)	0.530		
Smoke status (N *vs*. Y)	0.67 (0.23−1.64)	0.462		
Tumor Stage (IV *vs*. III)	2.59 (1.25−5.39)	**0.011**	2.76 (1.31−5.81)	**0.008**
Histology (AC *vs*. non-AC)	1.20 (0.69−2.08)	0.520		
EGFR Status (MT *vs*. WT)	0.90 (0.22−3.72)	0.889		
Sodium Normalization (N *vs*. Y)	1.81 (1.01−3.31)	**0.047**	1.96 (1.05−3.66)	**0.035**

Significant values are reported in bold.

AC = Adenocarcinoma; CI = confidence interval; ECOG-PS = Eastern Cooperative Oncology Group Performance Status; EGFR = Epidermal growth factor receptor; F = female; HR = hazard ratio; M = male; MT = mutated status; WT = wild-type status

Univariate analysis showed that adenocarcinoma histotype and sodium normalization were significantly associated with longer PFS (Table 3B). Multivariate Cox regression analysis confirmed the prognostic value of sodium normalization (Table 3B).

**Table 3B:** Univariate and multivariable analysis of predictors of PFS in the 69 patients with hyponatremia at the start of first-line therapy for locally advanced or metastatic NSCLC

PROGRESSION-FREE SURVIVAL IN HYPONATREMIC PATIENTS				
	Univariate Cox Regression		Multivariable Cox regression	
	HR (95%CI)	*p-value*	HR (95%CI)	*p-value*
Age (≥ 70y *vs*. < 70y)	0.91(0.44−1.85)	0.789		
Gender (F *vs*. M)	0.64 (0.30−1.36)	0.250		
ECOG-PS (≥ 2 *vs*. < 2)	1.53 (0.66−3.54)	0.322		
Smoke status (N *vs*. Y)	0.52 (0.15−1.74)	0.288		
Tumor Stage (IV *vs*. III)	1.55 (0.73−3.30)	0.259		
Histology (AC *vs*. non-AC)	0.56 (0.28−1.11)	**0.097**	0.70 (0.33−1.48)	0.359
EGFR Status (MT *vs*. WT)	0.62 (0.08−4.57)	0.641		
Sodium Normalization (N *vs*. Y)	2.61 (1.22−5.57)	**0.014**	2.22 (1.02−5.04)	**0.047**

Significant values are reported in bold.

AC = Adenocarcinoma; CI = confidence interval; ECOG-PS = Eastern Cooperative Oncology Group Performance Status; EGFR = Epidermal growth factor receptor; F = female; HR = hazard ratio; M = male; MT = mutated status; WT = wild-type status

## DISCUSSION

Hyponatremia is the most common electrolyte disorder encountered in cancer patients [22, 23, 24, 25].

Hyponatremia has been identified as a negative prognostic factor in a number of different malignancies[5, 9, 26, 27, 28, 29, 30, 31, 32]. In the lung cancer population hyponatraemia is a negative prognostic factor in hospitalized patients and those with advanced-stage disease treated with erlotinib [33, 34]. Furthermore it has been shown to negatively correlate with the performance status [20] as well as tumour status and inflammation in completely resected NSCLC [35]. It is important for physicians to determine and validate prognostic factors in order to optimize and personalize the management of NSCLC. Therefore we evaluated the prognostic value of hyponatremia in 433 NSCLC patients treated with first-line chemotherapy or targeted therapy. We observed a significant difference between eunatremic and hyponatremic patients in OS (15.5 *vs*. 8.8 months, respectively) but not PFS (6.3 *vs*. 4.1 months *p* = 0.24). Futhermore, for the first time, we showed the prognostic significance of hyponatremia normalization in patients with advanced NSCLC treated with first line therapy.

Our results are consistent with those reported in SCLC by Hansen *et al*. that showed that hyponatremia was associated with a lower median OS in a retrospective study of 453 SCLC patients undergoing chemotherapy. The study showed also that patients who did not fully correct serum sodium values within the first two cycles of chemotherapy had a worse outcome [25]. Hence, our results showed that the correction of sodium levels was associated with significantly higher OS (11.6 *vs*. 4.7 months) and PFS (6.7 *vs*. 3.3 months) in patients with NSCLC treated with first-line therapy. Lack of hyponatremia normalization was associated with worse OS and PFS at univariate and multivariate analyses. Thus suggesting that an early detection, a careful monitoring and supportive therapy of hyponatremia can help to improve the medical case and prognosis.

It is therefore important to achieve international consensus about the optimal investigation, diagnosis and management of hyponatraemia in order to optimize the outcome of NSCLC patients.

There are limitations to this study. First, it is a retrospective analysis, which is therefore susceptible to bias in data selection and analysis. A prospective study would be useful to validate these results.

Secondly, the management of hyponatremia was not standardized in all patients and therefore it is not possible to certain whether failure to normalize serum sodium was a reflection of the overall clinical scenario or sub-optimal medical management. Finally, concurrent drugs cannot be fully accounted for could influence the cause and course of hyponatremia.

Our results confirm the prognostic value of low serum sodium in NSCLC patients treated with first-line therapy and underline the importance of a prompt and effective correction of hyponatremia in lung cancer patients.

## MATERIALS AND METHODS

### Study population and data collection

The study population included adult patients with histologically or cytologically confirmed diagnosis of locally advanced or metastatic NSCLC treated with first-line chemotherapy or targeted therapy at two institutions (Università Politecnica Marche, Italy and Chelsea & Westminster Hospital, UK) between 1^st^ May 2006 and 31th January 2015. Tumor stage was assessed according to the tumor-node-metastasis (TNM) system and included patients with stage IIIB, IV and IIIA not suitable for surgery, as defined in AJCC version 7. Data were retrospectively collected from patients’ medical records.

Treatment with first-line chemotherapy or targeted therapy was continued until evidence of disease progression, unacceptable adverse events, or death. Follow-up generally consisted of regular physical examination and laboratory assessment (haematology and serum biochemistry), and imaging using computed tomography (CT) or magnetic resonance imaging (MRI) according to local procedures every 8-12 weeks.

Overall survival (OS) was defined as the time from beginning of first-line treatment to death, irrespective of cause. Progression free survival (PFS) was defined as the time from beginning of treatment to progression or to death from any cause, whichever occurred first. Patients without tumour progression or death at the time of the data cut-off for the analysis or at the time of receiving an additional anticancer therapy were censored at their last date of tumour evaluation.

### Statistical Analysis

PFS and OS were estimated using Kaplan-Meier method with Rothman's 95% confidence intervals (CI) and compared across the groups using the log-rank test. Patients with a stable disease (SD), partial remission, and a complete remission were considered as responders.

Hyponatremia was assessed within one week prior to starting first-line therapy, and after each treatment cycle. Potential factors associated with outcome were evaluated, including patients’ age (≥ 70y *vs*. < 70y), gender, tumor stage, histology, EGFR mutational status, Eastern Cooperative Oncology Group performance status (PS) and smoking history. Data about concomitant medications were not available.

Cox proportional hazards models were applied to explore patients’ characteristics predictors of survival in univariate- and multivariable analysis. Variables not fitting at univariate analysis were excluded from the multivariate model. No-multicollinearity of the grouped co-variates was checked. Significance level in the univariate model for inclusion in the multivariate final model was more liberally set at a 0.2 level [36, 37]. The likelihood ratio test was conducted to evaluate the improvement in prediction performance gained by backward elimination of variables from the prognostic model [38]. All other significance levels were set at a 0.05 value and all *P* values were two-sided. Statistical analyses were performed using MedCalc version 11.4.4.0 (MedCalc Software, Broekstraat 52, 9030 Mariakerke, Belgium). The research was carried out in accordance with the ethical committee of our institution. All patients gave their written consent to all the diagnostic-therapeutic procedures.
